# Supplementary material for: Association between Prehospital Hypoxemia and Admission to Intensive Care Unit during the COVID-19 Pandemic: A Retrospective Cohort Study
Source: Medicina (Kaunas). 2021 Dec 14;57(12):1362. doi: 10.3390/medicina57121362 (PMC8707267; doi:10.3390/medicina57121362)
Supplement: Supplementary file 1 [file medicina-57-01362-s001.zip › medicina-1491602-supplementary.pdf]

Table S1: ICU fast-track admission criteria (adapted from [4,11])

ICU admission criteria	Requirement for invasive ventilatory support<br>- prehospital intubation<br>- an arterial oxygen saturation (SaO_2) <90% on oxygen (O_2) with persistent signs of respiratory insufficiency.
Exclusion criteria	Patient's wishes (advance directive, etc.)
	Unwitnessed cardiac arrest, recurrent cardiac arrest, cardiac arrest with no return of spontaneous circulation
	Malignant disease with a life expectancy of less than 12 months
	End-stage neurodegenerative disease
	Severe and irreversible neurological event or condition
	Chronic condition:<br>- NYHA class IV heart failure<br>- COPD GOLD 4 (D)<br>- Liver cirrhosis, Child-Pugh score >8<br>- Severe dementia
	Severe circulatory failure, treatment-resistant despite increased vasoactive dose (hypotension and/or persistent inadequate organ perfusion)
	Estimated survival <12 months
